# Adequacy of Mental Health Services for HIV-Positive Patients with Depression: Ontario HIV Treatment Network Cohort Study

**DOI:** 10.1371/journal.pone.0156652

**Published:** 2016-06-09

**Authors:** Stephanie K. Y. Choi, Eleanor Boyle, John Cairney, Sandra Gardner, Evan J. Collins, Jean Bacon, Sean B. Rourke, OHTN Cohort Study Group

**Affiliations:** 1 The Ontario HIV Treatment Network, Toronto, Ontario, Canada; 2 The Institute of Medical Science, Faculty of Medicine, University of Toronto, Toronto, Ontario, Canada; 3 Institute of Sports Science and Clinical Biomechanics, University of Southern Denmark, Odense, Denmark; 4 Dalla Lana School of Public Health, University of Toronto, Toronto, Ontario, Canada; 5 Department of Clinical Epidemiology and Biostatistics, McMaster University, Hamilton, Ontario, Canada; 6 Centre for Addiction and Mental Health, Toronto, Ontario, Canada; 7 The Institute for Clinical Evaluative Sciences, Toronto, Ontario, Canada; 8 University Health Network, Toronto, Ontario, Canada; 9 Department of Psychiatry, Faculty of Medicine, University of Toronto, Toronto, Ontario, Canada; 10 St. Michael’s Hospital, Toronto, Ontario, Canada; 11 Baycrest Health Sciences, Toronto, Ontario, Canada; British Columbia Centre for Excellence in HIV/AIDS, CANADA

## Abstract

**Background:**

Major depression can profoundly impact clinical and quality-of-life outcomes of people living with HIV, and this disease is underdiagnosed and undertreated in many HIV-positive individuals. Here, we describe the prevalence of publicly funded primary and secondary mental health service use and antidepressant use, as well as mental health care for depression in accordance with existing Canadian guidelines for HIV-positive patients with depression in Ontario, Canada.

**Methods:**

We conducted a prospective cohort study linking data from the Ontario HIV Treatment Network Cohort Study with administrative health databases in the province of Ontario, Canada. Current depression was assessed using the Center for Epidemiologic Depression Scale or the Kessler Psychological Distress Scale. Multivariable regressions were used to characterize prevalence outcomes.

**Results:**

Of 990 HIV-positive patients with depression, 493 (50%) patients used mental health services; 182 (18%) used primary services (general practitioners); 176 (18%) used secondary services (psychiatrists); and 135 (14%) used both. Antidepressants were used by 407 (39%) patients. Patients who identified as gay, lesbian, or bisexual, as having low income or educational attainment, or as non-native English speakers or immigrants to Canada were less likely to obtain care. Of 493 patients using mental health services, 250 (51%) received mental health care for depression in accordance with existing Canadian guidelines.

**Conclusions:**

Our results showed gaps in delivering publicly funded mental health services to depressed HIV-positive patients and identified unequal access to these services, particularly among vulnerable groups. More effective mental health policies and better access to mental health services are required to address HIV-positive patient needs and reduce depression’s impact on their lives.

## Introduction

Major depression is a substantial burden to HIV-positive patients, and the mental health needs of these patients are frequently unmet. There is evidence that depression is under-diagnosed and under-treated in HIV-positive patients [1,2]. Approximately half of these patients do not receive treatment [3–6]. Studies have shown that untreated depression is associated with poor antiretroviral therapy (ART) compliance [7–9], poor clinical and quality-of-life outcomes [10,11], increased comorbidity and mortality [10,11], and elevated HIV transmission risk [12–15]. Because HIV-positive patients increasingly live longer, these negative effects are likely to have long-term impacts, making effective mental health care essential.

Before we can provide effective mental health care for HIV-positive patients, we must understand how the patients use mental health services currently. The U.S. evidence showed that approximately 40% of HIV-positive patients discuss mental health concerns with their family physicians, 20–39% seek help from mental health specialists (e.g., psychiatrists, mental health nurses, psychologists, and psychotherapists), and 20–54% use antidepressants [3,5,6,16–19]. However, most evidence is based on older data collected from the US between 1996 and 1997, so this might not reflect current needs in the HIV-positive community [5,16–19]. Additionally, this evidence focused on examining access to mental health care in HIV-positive patients in general instead of examining those with psychiatric disorders or perceived needs, the gaps in access to mental health care could be overstated [16–19]. There are three U.S. studies provided further information for HIV-positive patients with perceived needs in mental health care. Patients who perceived a need for care were two to three times more likely to seek help from mental health care providers [5,6], and 38–46% of HIV-positive women with depression received depression treatments that met evidence-based guidelines [3]. However, these studies were limited by self-report mental health services data [5,6], solely focusing on female HIV-positive patients [3], or solely focusing in patients with severe co-occurring mental and addiction disorders [6].

To our knowledge, there are no comparable studies of mental health service use by HIV-positive individuals in Canada. Because of differences between the U.S. and Canadian health care systems, the evidence from the U.S. may not be directly applicable for program planners, policy-makers, and clinicians trying to deliver effective mental health care to Canadian HIV-positive patients. Currently, the Canadian mental health system is undergoing a series of reforms [20]. However, reforms take time to implement, and many challenges need to be addressed, including: the lack of coordination and integration among different parts of the mental health care system [20–22]; inadequate resources and support for family physicians [23]; long wait times for psychiatrists [24]; service access that is difficult to navigate [21]; disparities in care delivery [21]; and inadequate resources and funding [25]. More concerning is that these challenges in accessing mental health care could be amplified in people living with HIV.

To address these knowledge gaps, in this study, we had three objectives: (1) to determine the 12-month prevalence of mental health service and antidepressant use among HIV-positive patients with current depression; (2) to examine the 12-month prevalence of mental health services in accordance with existing Canadian guidelines [26–28]; and (3) to characterize the impact of individual-level predictors (i.e., predisposing factors, enabling factors, and need) on these prevalence outcomes.

## Materials and Methods

We conducted a multi-center prospective cohort study between October 1, 2007 and March 31, 2013 using data collected from the Ontario HIV Treatment Network Cohort Study (OCS) and the Ontario Ministry of Health and Long-Term Care (MOHLTC). Ethics approval was granted by all of the institutions involved (i.e., the Institutional Review Board at Sunnybrook Health Sciences Centre, Ottawa Health Science Network Research Ethics Board, The University of Western Ontario Research Ethics Board for Health Sciences Research involving Human Subjects, St. Michael's Hospital Research Ethics Board, the Research Ethics Board of Health Sciences North, Sunnybrook Health Sciences Centre Research Ethics Board, University Health Network Research Ethics Board, and Windsor Regional Hospital Research Ethics Board).

### Data Sources

The OCS was linked to a number of administrative health databases maintained by the Institute for Clinical Evaluative Sciences (ICES) using uniquely encoded identifiers. The OCS is one of the largest HIV medical cohorts in North America. It has followed HIV-positive patients receiving health care from twelve HIV clinics across Ontario since 1995. At the time of our study, HIV-positive study participants were recruited from 12 participating OCS sites, which are mainly hospital-based speciality HIV clinics, hospital-based family practice units, and community-based primary care physician practices across Ontario [29]. A full description of the OCS has been published previously [29].

Our analysis period began on October 1, 2007 when additional questions were added to the standard OCS questionnaire that measured participants’ depression status, social demographics, HIV clinical markers, and psychosocial behaviours. Clinical nurses and assistants interviewed participants during regular clinical appointments [29]. Most OCS participants completed follow-up interviews annually. Due to resource constraints, seven suburban clinics outside Toronto used a “brief” version of the questionnaire (30 minutes) that relied on the 10-item Kessler Psychological Distress Scale (K_10_) to determine depression. The remaining clinics used a “full” version (120 minutes) that relied on the 20-item Centre for Epidemiologic Depression Scale (CES-D_20_).

In Canada, the publicly funded universal health care system covers medical and hospital services provided by physicians for insured residents of Canada. We used three administrative databases and the OCS medication database to obtain participants’ primary and secondary medical mental health service use, their antidepressant use, their previous depression-related condition diagnoses, and their death records. The databases are intended for administrative purposes but they have been adopted widely for health service and policy research in Ontario [30]. In Ontario, all billing records for publicly funded physician services are collected in administrative databases. The Ontario Health Insurance Plan (OHIP) database contains billing records for all insured mental health service claims submitted by physicians and psychiatrists. The Ontario Drug Benefit (ODB) formulary database contains records of publicly funded antidepressant medications dispensed to OCS participants who are ≥65 years of age, receive social assistance, or have high prescription drug costs. Approximately 65% of OCS participants had ODB records during our study period. We used both ODB records and medications listed in clinical records of the OCS medication database to identify antidepressant use. The Registered Persons Database (RPDB) contains death records for all OCS participants who were insured under OHIP at the time of their death. Death records reported in the RPDB are generally accurate compared with Ontario Vital Statistics data [31]. (More details about the administrative databases can be found at http://datadictionary.ices.on.ca.)

### Cohort Enrolment

**Fig 1:**
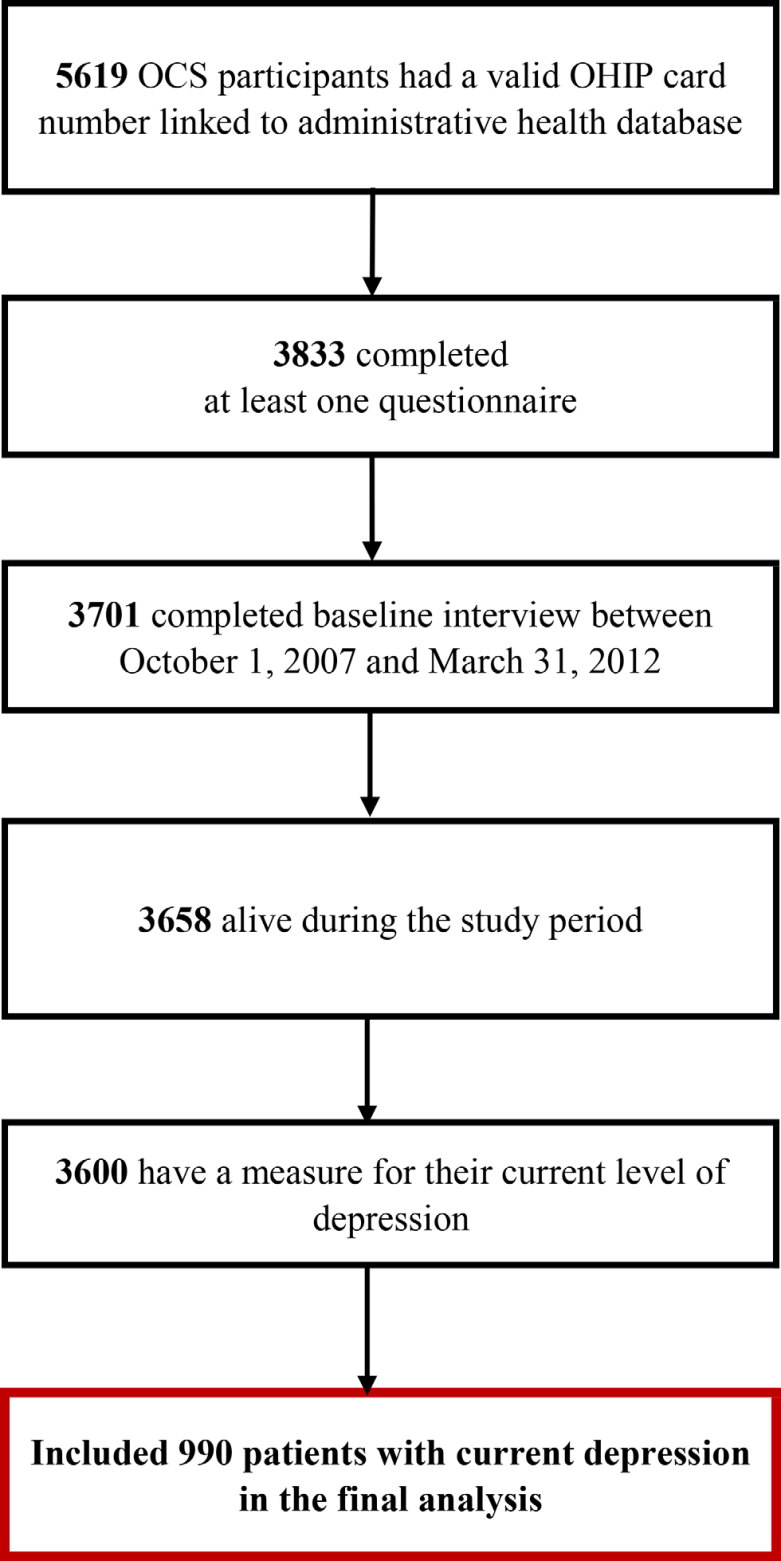
Patient Flow Chart for Development of Prevalence, Persistence and Incidence Cohort.

Fig 1 shows the cohort assembly for this study. Between October 1, 2007 and December 31, 2012, clinical nurses at each OCS study site administered questionnaires to OCS participants during their annual appointment. The participant baseline was defined as the date when he or she completed the first interview. Each participant was followed for 1 year after baseline.

Initially, we included 3,701 participants who completed a baseline interview and had a valid OHIP number. We excluded 43 participants who died within 1 year following the baseline assessment. We also excluded 58 participants who were not screened for depression at baseline.

We assessed the patient’s current self-reported depression using either the CESD_20_ or the K_10_. Sixty-one percent of the OCS participants received the K_10_ and the rest received the CESD_20_. The diagnostic accuracy and reliability of the CESD_20_ and K_10_ for identifying depression were previously assessed using DSM-IV criteria for a diagnosis of major depression in a sample of OCS participants [32]. The CESD_20_ has a sensitivity of 1.0 and a specificity of 0.88; the cut-off value used to identify participants with current depression was 23 [32]. The K_10_ has a sensitivity of 0.95 and a specificity of 0.82; the cut-off value used to identify participants with current depression was 22 [32]. The CESD_20_ and K_10_ demonstrate good inter-rater agreement (Cohen’s Kappa Statistic = 0.79) when compared with the DSM-IV criteria for a diagnosis of major depression [32].

### Measures

In Ontario, most of mental health care is delivered by general physicians or family physicians (primary mental health services) or psychiatrists (secondary mental health services) and covered by OHIP [33,34]. General and family physicians act as gatekeepers, referring patients to specialty care provided by psychiatrists [21,33].

We examined three measures for the use of physician-provided mental health services in Ontario:

1. Primary mental health services were defined using a validation algorithm that combined mental health service codes and mental disorder diagnostic codes from the OHIP databases. This algorithm has high sensitivity (81%) and specificity (97%) [35].
2. Secondary mental health services were defined using the OHIP database and were based on the service code submitted by psychiatrists (specialty code = 19).
3. Antidepressant use was based on the first line of antidepressants for managing depression in adults recommended by the Canadian Network for Mood and Anxiety Treatments (CANMAT) Clinical guidelines [36]: (a) *selective serotonin reuptake inhibitors*, including fluovoxamine, citalopram, escitalopram, sertraline, fluoxetine, and paroxetine; (b) *serotonin and norepinephrine reuptake inhibitors*, including venlafaxine, duloxetine, desvenlafaxine, and milnacipran; and (c) *noradrenergic and specific serotonergic antidepressants*, including mirtazapine and mianserin; (d) other first-line antidepressants, including agomelatine, bupropion, moclobemide, reboxetine, and tianeptine.
4. We evaluated if the patients received mental health care for depression in accordance to existing Canadian guidelines [26–28], which specify (a) at least four visits to primary or secondary mental health service providers plus at least 2 months of antidepressant use or (b) at least eight visits to primary or secondary mental health service providers with a minimum visit time of 20 minutes. For each measure, we determined the 12-month prevalence rate.

### Predisposing, Enabling, and Need Factors

Using Andersen and colleagues’ behavioural framework for health services use [37], we examined individual-level determinants for mental health service use and the receipt of mental health care for depression in accordance with existing Canadian guidelines. We focused on three groups of individual-level predictors: predisposing factors, enabling factors, and need. Predisposing factors are socio-demographic and contextual characteristics that predispose individuals to use available services. Enabling factors are financial and organizational characteristics that allow individuals to use services. Need includes both perceived (i.e., an individual’s assessment of his or her health) and evaluated needs (i.e., a professional assessment). More information about variable measurements is provided in Table 1.

**Table 1:** Measurements of Predisposing, Enabling, and Need Predictors.

Variables	Categories	Measuring Instruments
**Predisposing Factors**		
Age	16–29, 30–39, 40–49, ≥50 years	Derived from birth date and interview date
Sex	Male, female	Self-reported
Sexual orientation	Yes or No	Self-reported as gay, bisexual, or lesbian
Ethnicity	Aboriginal, African/Caribbean, Asian, Latin American, English or other European	Self-reported
Canadian Immigrant	Yes or No	Self-reported
Employment status	Employed (reference group), unemployed or not in workforce, current recipients of Ontario Disability Support Program	Self-reported
Completion of high school or less	Yes or No	Self-reported
Married or living with partner	Yes or No	Self-reported
**Enabling Factors**		
Annual gross household income before withholding taxes	<$20K, $20K-$39,999, $40K-$49,999, ≥$50K CAD	Self-reported
Difficulty in affording house-related expenses	Yes or No	A 5-point Likert scale (strongly agree to strongly disagree) in response to: “Considering your household income, how difficult is it for you to meet your monthly housing-related costs?”
**Need Factors**		
Past history of depression	Yes or No	Past depression diagnoses were defined as having past depression-related diagnosis identified in OHIP records (OHIP ICD-9: 296 and 311), from the earliest available records to a year before the baseline.
Current smoker	Yes or No	Self-reported
Prior diagnosis of alcohol abuse	Yes or No	Addiction to alcohol was defined when patients had a diagnostic code of alcohol dependence/abuse in OHIP (ICD-9: 303) or in main diagnosis of DAD and NACRS (ICD-9-CM: 303; ICD-10-CA: F10), from the earliest available records in these databases to a day before the baseline.
Recreational drug use (in past 6 months)	Yes or No	Self-reported
CD4 cell count (in past 6 months)	Yes or No	Yes if CD4 cell counts less than 200 mL from recent HIV antibody tests during past 6 months from each interview date.
Non-suppressed viral loads (in past 6 months)	Yes or No	Yes if non-suppressed viral loads (>50 mL) from recent HIV antibody tests during past 6 months from each interview date.
Years since HIV diagnosis	Continuous variable	Derived from the interview date and the date of HIV diagnosis.
Physical quality-of-life	Continuous variable: range of 0–100	Twelve-item short form health survey version 2 (SF-12v2) ^a^^,^^b^.
Mental quality-of life	Continuous variable: range of 0–100	Twelve-item short form health survey version 2 (SF-12v2) ^a^^,^^b^.
Multi-morbidity	0, 1+	Yes if score was greater than 1 on the Charlson-Deyo comorbidity index^c^.

^a^ Source: Ware, Kosinski, & Keller, 1996

^b^ Source: Chariyalertsak et al., 2011

^c^ Source: Deyo, Cherkin, & Ciol, 1992

### Statistical Analyses

The baseline characteristics of the sample were described using frequency and proportion for categorical variables, and using mean and standard deviation for continuous variables. The 12-month prevalence of the three mental health service utilization measures (i.e., primary mental health service use, secondary mental health service use, and antidepressant use) were estimated based on the 1-year follow-up period. To determine whether the patients received mental health care for depression in accordance with existing Canadian guidelines, we estimated the 12-month prevalence of those individuals who had at least one visit with primary or secondary mental health care providers.

Multivariable logistic regression models were constructed to examine associations between the three groups of individual-level predictors (predisposing, enabling, and need-related factors) and mental health service use or the receipt of mental health care for depression in accordance with existing Canadian guidelines. For the receipt of mental health care for depression (according to existing guidelines), we constructed multivariable regression models for patients who had at least one visit with primary or secondary mental health care providers. We selected covariates based on those described previously in the literature [3,5,6,16–19]. Those variables not described in the literature were selected based on a p value less than 0.25 for the crude association between the measure and each predictor [38]. Covariates were only removed from final models if their p value was greater than 0.2 to prevent residual confounding [38,39]. The final sets of covariates were selected based on the results of likelihood ratio tests using nested models or Akaike or Bayesian information criteria [38]. All final models were also controlled for questionnaire type (full vs. brief). We reported the adjusted odds ratio (aOR) and corresponding 95% confidence intervals (CIs) for the associations.

We also conducted several model diagnostics on our final models [40]. We assessed model fit using goodness-of fit measures. Multi-collinearity was examined using a variance inflation factor. Influential observations were assessed using leverage and Pregibon’s dbeta statistics. Continuous predictor linearity was assessed using partial-residual plots. The results indicated that our final models were robust.

All statistical analyses were performed using STATA MP version 13.1 [41] and were conducted at the ICES. A two-sided statistical significance was determined by a p value less than 0.05.

## Results

The prevalence of current depression was 28% (95% CI: 26–29%; 990 of 3600 patients assessed). Of the 990 depressed HIV-positive patients, the mean age was 44 years (SD = 9), and 22% were female. Baseline characteristics of our sample are described in Table 2. Briefly, 551 patients (56%) had a history of depression (determined using OHIP diagnostic codes until 1 year prior to baseline). The mean scores for physical and mental health-related quality of life were 45 (SD = 12) and 33 (SD = 10), respectively. Depressed patients primarily self-reported as gay, lesbian, or bisexual (61%), recipients of the Ontario Disability Support Program (ODSP) (54%), or current smokers (54%). Forty percent were living below the poverty line (i.e., gross household income <$20,000 CAD per year).

**Table 2:** Baseline Characteristics of the Sample (N = 990).

Characteristics	Total	
	(N = 990)	
	n	%
**Demographics**		
Age		
16–29 years	74	(7%)
30–39 years	226	(23%)
40–49 years	434	(44%)
≥ 50 years	256	(29%)
Female	220	(22%)
Gay, lesbian, or bisexual	605	(61%)
Married / living with partner	309	(31%)
Ethnic identity		
First Nation, Metis, or Inuit	132	(13%)
African, Caribbean, Asian, or Latin American	189	(19%)
British (or English, Irish, Welsh or Scottish)	283	(29%)
All Other European descent	386	(39%)
Canadian immigrant	282	(28%)
Non-English spoken at home	123	(12%)
**Socio-economic Status**		
Current Employment status		
Unemployed	133	(13%)
Student/Retired	48	(5%)
Receipt of Ontario Disability of Subsidies Program	531	(54%)
Employed	271	(27%)
Completed high school or less	412	(42%)
Annual household income (CAD) before withholding taxes/benefits		
< $20,000	386	(39%)
$20,000 to $39,999	212	(21%)
$40,000 to $49,999	89	(9%)
≥ $50,000	186	(19%)
Difficulty in affording housing-related expenses^a^	183	(18%)
**Harmful Behaviors**		
Recreational drug use in past 6 months	292	(29%)
Prior diagnosis of alcohol abuse^b^	178	(18%)
Current smokers	530	(54%)
**Health Status**		
Physical component scale of SF12, mean(SD)	45	(12)
Mental component scale of SF12, mean(SD)	33	(10)
Charlson co-morbidity index ≥ 1^c^	305	(31%)
CD4 cell count < 200 *μL* (in past 6 months)	114	(12%)
Non-suppressed viral load (> 50 *μL*) (in past 6 months)	339	(34%)
Years since HIV diagnosis, mean (SD)	11	(7)
Prior diagnosis of depression	551	(56%)
**Questionnaire Type**^**d**^		
Brief	698	(71%)
Full	292	(29%)

^a^ Difficulty in affording house-related expenses was defined as a patient’s self-reported “Very difficult” or “Fairly difficult” for the following question: “Considering your household income, how difficult is it for you to meet your monthly housing-related costs?(Housing costs include rent/mortgage, property taxes and utilities only).”

^b^ Addiction to alcohol was defined when patients had a diagnostic code of alcohol dependence/abuse in OHIP (ICD-9: 303) or in the main diagnosis of DAD and NACRS (ICD-9-CM: 303; ICD-10-CA: F10), from the earliest available records in these databases to a day before baseline.

^c^ Multi-morbidity was measured by the Charlson-Deyo comorbidity index. A score of greater than 1 indicates the presence of comorbidities. A score of zero indicates no comorbidities.

^d^ There are two structural questionnaires administered annually by clinic nurses during the patient’s regular clinical appointments. Due to constraints on human resources and time, seven clinics have adopted a “brief” version of the questionnaire. Full details of the cohort can be found on the study website: http://www.ohtncohortstudy.ca/.

### Twelve-Month Prevalence of Mental Health Services Use

Of 990 HIV-positive patients with depression, 493 (50%) used primary and secondary mental health services. Specifically, 182 (18%) used primary mental health services alone, 176 (18%) used secondary mental health services alone, and 135 (14%) used both primary and secondary service providers. Four hundreds and seven (41%) patients used antidepressants. Among 493 patients who had at least one visit with a primary or secondary mental health care provider, 250 (51%) received mental health care for depression in accordance with existing Canadian guidelines.

### Determinants of Mental Health Service Use and Antidepressant Use

**Fig 2:**
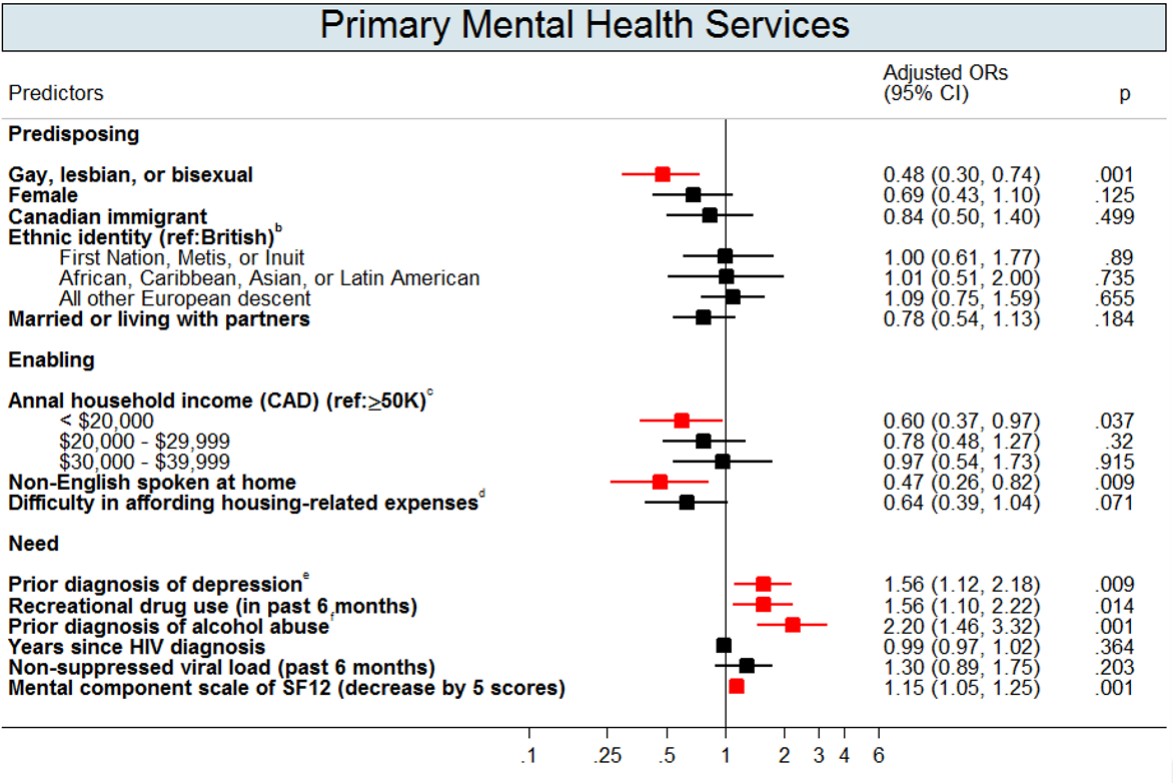
Adjusted Odds Ratios (OR) with 95% Confidence Intervals (CIs) for the Predictors of Primary Mental Health Services Utilization^a^. Footnotes: This graph contains the final set of covariates retained in the multivariable logistic regression model for primary mental health services use outcome. A red colour dot indicates an adjusted odds ratio with a p-value < 0.05; a black color dot indicates an adjusted odds ratios with a p-value ≥ 0.05. ^a^ Primary mental health services (provided by general or family physicians) were defined by a validation algorithm (Steele et al., 2004) using mental health service codes and a mental disorder diagnostic code from Ontario Health Insurance Program (OHIP) database. ^b^ Aboriginals were participants who self-reported as Aboriginals, North American Indian, Firsts Nations, Metis, or Inuit. African, Caribbean, Asian, or Latin American were participants who self-reported as African, Caribbean, Chinese, South Asian, or Latin American. English were participants who were self-reported as English, Sottish, Irish, or Welsh. All other European descent were participants who self-reported as Polish, French, Portuguese, German, Norweigan, Italian, Swedish, Ukranian, Dutch, or Jewish. ^c^ Gross income before taxes and benefits. ^d^ Difficulty in affording house-related expenses was defined as when the patients self-reported as being very difficult or fairly difficult to meet their monthly housing-related costs (includes rent/mortgage, property taxes and utilities only). ^e^ Past depression diagnoses were defined as having a past depression-related diagnosis identified in OHIP records (OHIP ICD-9: 296 and 311), from the earliest available records to a year before the baseline. ^f^Addiction to alcohol was defined as whether patients had a diagnostic code of alcohol dependence/abuse in OHIP (ICD-9: 303) or in main diagnosis of DAD and NACRS (ICD-9-CM: 303; ICD-10-CA: F10), from the earliest available records in these databases to a day before the baseline.

**Fig 3:**
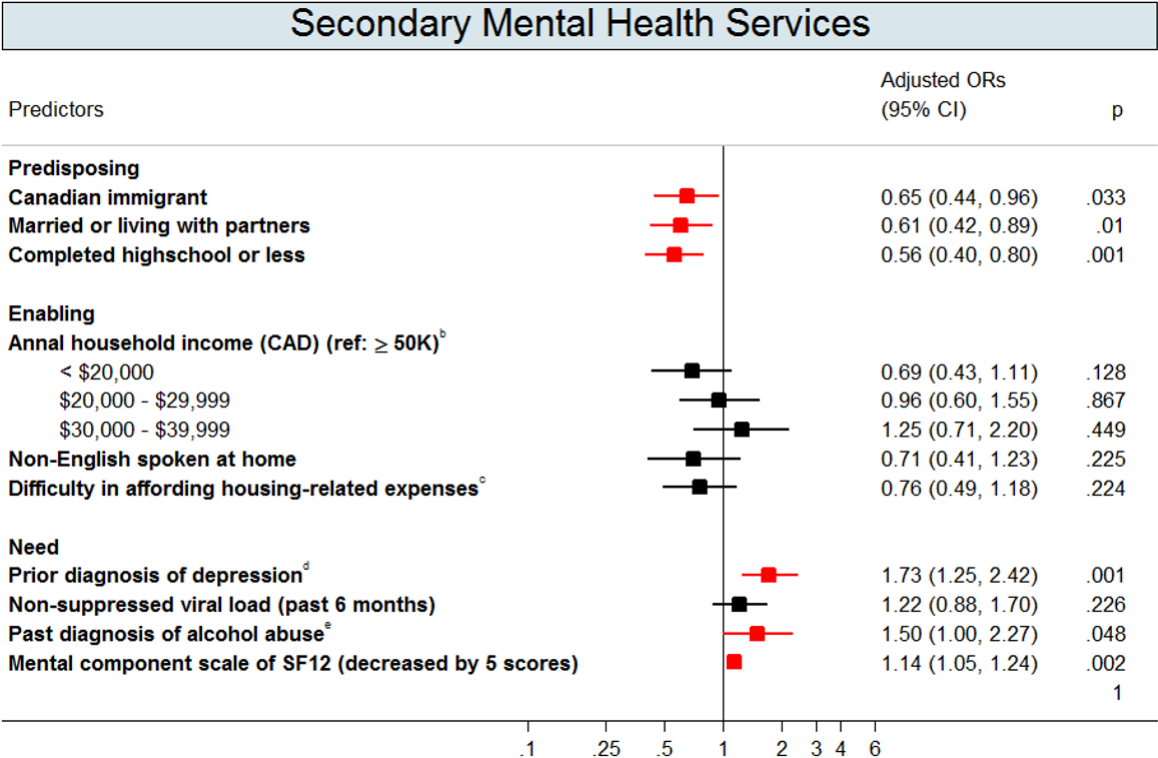
Adjusted Odds Ratios (OR) with 95% Confidence Intervals (CIs) for the Predictors of Secondary Mental Health Services Utilization^a^. Footnotes: This graph contains the final set of covariates retained in the multivariable logistic regression model for secondary mental health services use outcome. A red colour dot indicates an adjusted odds ratio with a p-value < 0.05; a black color dot indicates an adjusted odds ratios with a p-value ≥ 0.05. ^a^ Secondary mental health services (provided by psychiatrists) were identified by a specialty code of 19 from OHIP database. ^b^ Gross income before taxes and benefits. ^c^ Difficulty in affording house-related expenses was defined as when the patients self-reported as being very difficult or fairly difficult to meet their monthly housing-related costs (includes rent/mortgage, property taxes and utilities only). ^d^ Past depression diagnoses were defined as having a past depression-related diagnosis identified in OHIP records (OHIP ICD-9: 296 and 311), from the earliest available records to a year before the baseline. ^e^ Addiction to alcohol was defined as whether patients had a diagnostic code of alcohol dependence/abuse in OHIP (ICD-9: 303) or in main diagnosis of DAD and NACRS (ICD-9-CM: 303; ICD-10-CA: F10), from the earliest available records in these databases to a day before the baseline.

**Fig 4:**
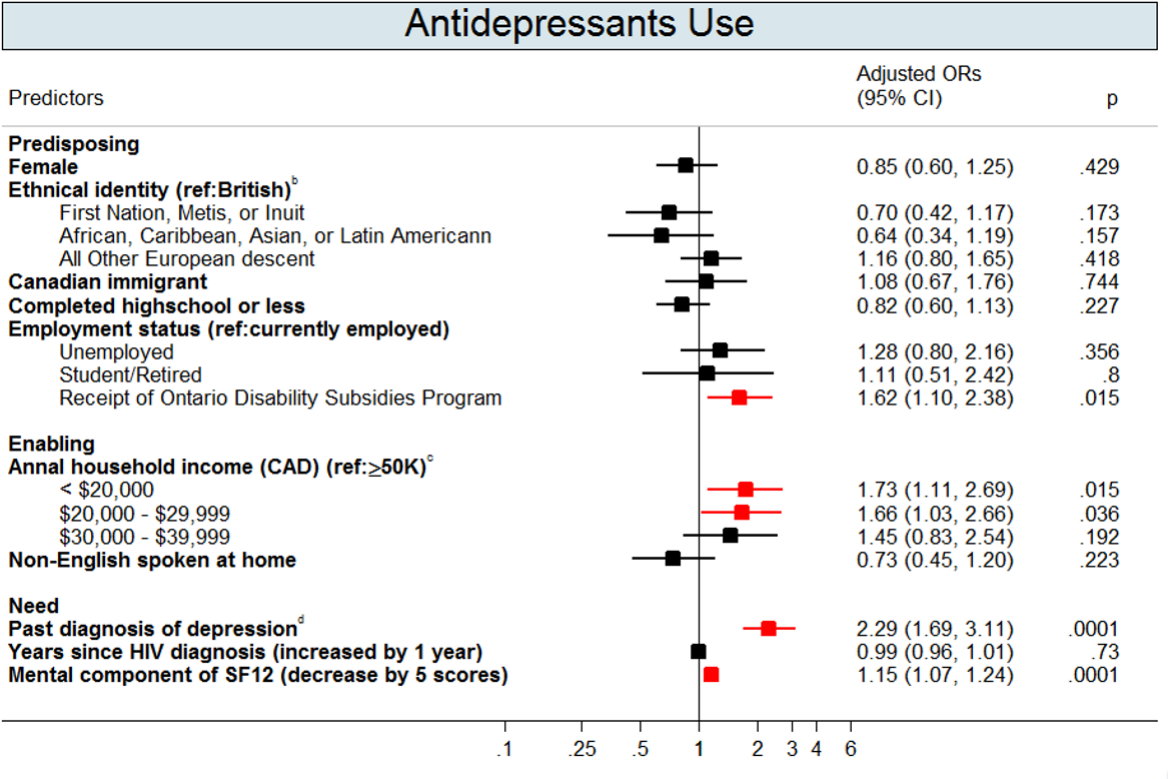
Adjusted Odds Ratios (OR) with 95% Confidence Intervals (CIs) for the Predictors of Antidepressant Use^b^. Footnotes: This graph contains the final set of covariates retained in the multivariable logistic regression model for antidepressant use outcome. A red colour dot indicates an adjusted odds ratio with a p-value < 0.05; a black color dot indicates an adjusted odds ratios with a p-value ≥ 0.05. ^a^ Antidepressant use was based on the first line of antidepressants for managing depression in adults recommended by the Canadian Network for Mood and Anxiety Treatments (CANMAT) Clinical guidelines (Lam et al., 2009): (a) selective serotonin reuptake inhibitors, including fluovoxamine, citalopram, escitalopram, sertraline, fluoxetine, and paroxetine; (b) serotonin and norepinephrine reuptake inhibitors, including venlafaxine, duloxetine, desvenlafaxine, and milnacipran; and (c) noradrenergic and specific serotonergic antidepressants, including mirtazapine and mianserin; (d) other first-line antidepressants, including agomelatine, bupropion, moclobemide, reboxetine, and tianeptine. ^b^ Aboriginals were participants who self-reported as Aboriginals, North American Indian, Firsts Nations, Metis, or Inuit. African, Caribbean, Asian, or Latin American were participants who self-reported as African, Caribbean, Chinese, South Asian, or Latin American. English were participants who were self-reported as English, Sottish, Irish, or Welsh. All other European descent were participants who self-reported as Polish, French, Portuguese, German, Norweigan, Italian, Swedish, Ukranian, Dutch, or Jewish. ^c^ Gross income before taxes and benefits. ^d^ Past depression diagnoses were defined as having a past depression-related diagnosis identified in OHIP records (OHIP ICD-9: 296 and 311), from the earliest available records to a year before the baseline.

Figs 2–4 show the multivariate logistic regressions of factors associated with mental health service use and antidepressant use during the 1-year follow-up period. Analyses of patient characteristics revealed barriers to accessing both mental health services and antidepressants. Patients who were gay, lesbian, or bisexual (aOR: 0.5; 95% CI: 0.3–0.7), were non-native English speakers (aOR: 0.5; 95% CI: 0.3–0.8), or had annual household incomes of <$20,000 CAD (aOR: 0.6; 95% CI: 0.4–0.96) were 41–51% less likely to use primary mental health services. People who were married or living with a partner (aOR: 0.6; 95% CI: 0.4–0.9), those who had not completed basic education only (aOR: 0.6; 95% CI: 0.4–0.8), or those who were Canadian immigrants (aOR: 0.7; 95% CI: 0.4–0.96) were 35–44% less likely to access secondary mental health services. Depressed patients with annual gross household income below $39,999 CAD were approximately twice as likely to use antidepressants compared to their higher income counterparts. Depressed patients who were disabled (aOR: 1.6; 95% CI: 1.1–2.4) were also more likely to use antidepressants when compared to those who were not.

In addition to predisposing and enabling factors, need significantly predicted mental health service use. Depressed patients with previous depression diagnoses were 2–3 times more likely to use primary (aOR: 1.6; 95% CI: 1.1–2.2) and secondary (aOR: 1.7; 95% CI: 1.3–2.4) mental health services and to use antidepressants (aOR: 2.3; 95% CI: 1.7–3.1). Additionally, depressed patients who perceived themselves as having a poor mental health-related quality of life were more likely to use mental health services and antidepressants. Patients who were recreational drug users (aOR: 1.6; 95% CI: 1.1–2.2) or who had a past alcohol addiction diagnoses (aOR: 2.2; 95% CI: 1.4–3.3) were approximately two times more likely to use primary mental health services than patients without substance abuse issues.

### Determinants of Receiving Mental Health Care for Depression in Accordance with Existing Canadian Guidelines

**Fig 5:**
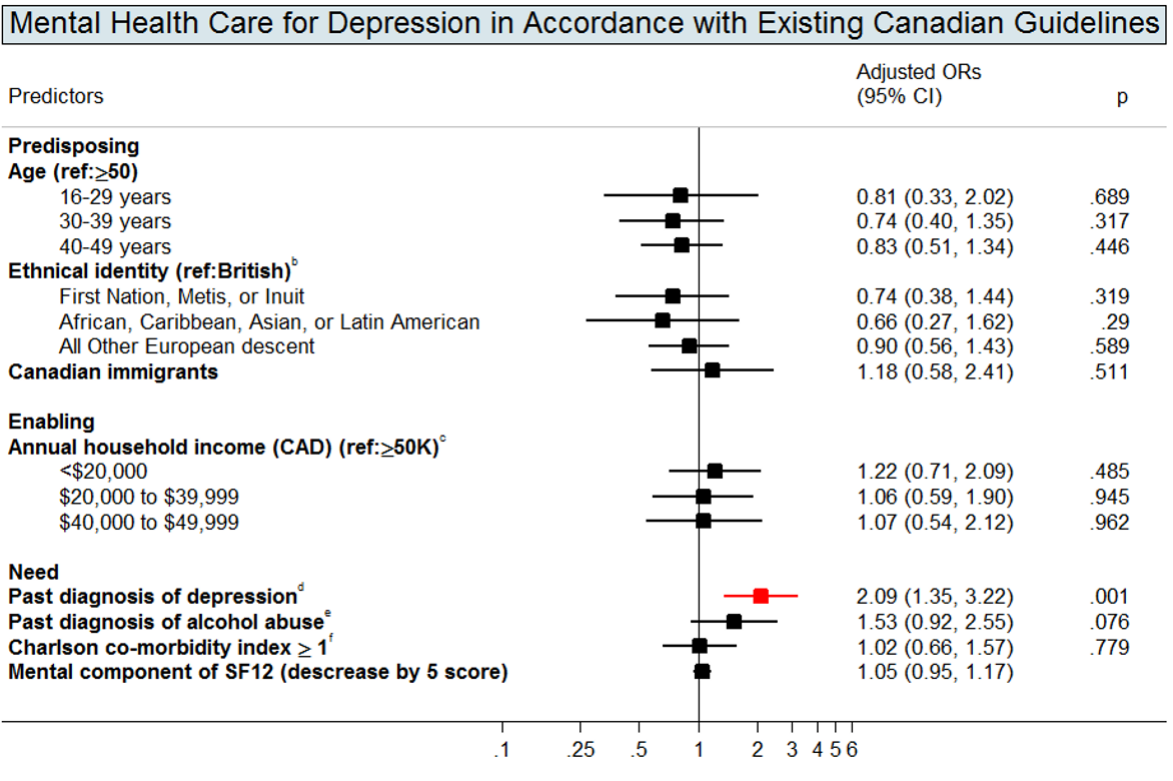
Adjusted Odds Ratios (OR) with 95% Confidence Intervals (CIs) for the Predictors of Receipt of Mental Health Care for Depression in Accordance with Existing Canadian Guidelines^d^. Footnotes: This graph contains the final set of covariates retained in the multivariable logistic regression model for the use of mental health care for depression in accordance with existing Canadian guidelines. We conducted the analysis only among those individuals who had at least one visit with primary or secondary mental health care providers. A red colour dot indicates an adjusted odds ratio with a p-value < 0.05; a black color dot indicates an adjusted odds ratios with a p-value ≥ 0.05. ^a^ Mental health care for depression in accordance with existing Canadian guidelines was based on two definitions according to Canadian guidelines: (i) at least two months of antidepressants use plus at least four visits to primary or secondary mental health services, OR (ii) at least eight visits to primary or secondary mental health services with patients spending at least 20 minutes with the GP/FP or psychiatrists (Canadian Network for Mood and Anxiety Treatments, 2001; Pattern et al., 2009; Ramasubbu et al., 2012). ^b^ Aboriginals were participants who self-reported as Aboriginals, North American Indian, Firsts Nations, Metis, or Inuit. African, Caribbean, Asian, or Latin American were participants who self-reported as African, Caribbean, Chinese, South Asian, or Latin American. English were participants who were self-reported as English, Sottish, Irish, or Welsh. All other European descent were participants who self-reported as Polish, French, Portuguese, German, Norweigan, Italian, Swedish, Ukranian, Dutch, or Jewish. ^c^ Gross income before taxes and benefits. ^d^ Past depression diagnoses were defined as having a past depression-related diagnosis identified in OHIP records (OHIP ICD-9: 296 and 311), from the earliest available records to a year before the baseline. ^e^ Addiction to alcohol was defined as whether patients had a diagnostic code of alcohol dependence/abuse in OHIP (ICD-9: 303) or in main diagnosis of DAD and NACRS (ICD-9-CM: 303; ICD-10-CA: F10), from the earliest available records in these databases to a day before the baseline. ^f^ Multi-morbidity was measured by Charlson-Deyo comorbidity index identified by inpatient discharge records over past five years before baseline (Deyo, Cherkin & Ciol, 1992). A score of greater than 1 indicates the presence of comorbidities; a score of zero indicates no comorbidities.

Fig 5 shows multivariate logistic regression analyses of factors predicting mental health care for depression in accordance with existing Canadian guidelines in 493 patients who had at least one visit with a primary or secondary mental health care provider. We did not find any predisposing or enabling factors associated with the receipt of mental health care for depression in accordance with existing Canadian guidelines. Depressed HIV-positive patients with past depression diagnoses were more likely to receive mental health care for depression in accordance with existing Canadian guidelines.

## Discussion

To our knowledge, this is the first study describing mental health service use by depressed HIV-positive patients in Canada and the first to compare the usage of mental health care in accordance to existing Canadian guidelines for depression care. Our analyses show that there are delivery gaps in mental health care for depressed HIV-positive patients. Only one-half of depressed HIV-positive patients use mental health services. Of those who use services, only one-half receive mental health care for depression in accordance with existing Canadian guidelines. We observe unequal access to publicly funded mental health services. Patients who were gay, lesbian, or bisexual, were immigrants to Canada, had low gross household incomes, had basic education only, or were non-native English speakers were less likely to access care.

Our reported prevalence for mental health service use (50%) was consistent with U.S. studies of HIV-positive patients (20–54%) [5,16–18] and the general population in Canada (53%) [42]. Additionally, the prevalence estimate of receipt of mental health care for depression in accordance with existing Canadian guidelines (51%) is comparable to estimates among HIV-positive women in the US (38–46%) [3] and the general population in Canada (55%) [43].

We identified several factors among HIV-positive patients with depression that were associated with a reduction in the use of mental health services. First, depressed patients who self-identified as gay, lesbian, or bisexual were 50% less likely to use primary mental health services. Although prior U.S. studies did not identify this association [3,5,6], our data are consistent with recent findings indicating a high prevalence of unmet mental health needs among gay, lesbian, bisexual or transgender individuals in Ontario, Canada [44]. In Ontario, McIntyre, Daley, Rutherford, & Ross (2012) conducted semi-structured interviews with eight Ontario mental health service providers with substantive experience with the lesbians, gays, bisexual, or transgender (LGBT) population and attempted to examine barriers in the current mental health care system in relation to providing care for this population [45]. The research revealed that there was a lack knowledge about LGBT-related issues among health care providers, and this may due to the fact that there is a lack of training around mental health services for the LGBT population in medical education [45]. Therefore, there are an inadequate number of trained mental health providers who can address issues among the LGBT population, resulting in long wait times for mental health services for this population [45]. A recent systematic review that included 14 studies (8 quantitative and 6 qualitative) revealed that many LGBT individuals have difficulties in accessing care due to communication difficulties with health care providers, difficulties disclosing their sexual orientation in the health care setting, institutionalized homophobia, and internalized homophobia [46]. Additionally, these individuals are more likely to discontinue mental health care due to negative experiences [44].

Second, we observed that low-income patients were less likely to use primary mental health services and patients with basic education only were less likely to use secondary mental health services. Unequal access to publicly funded mental health services provided by physicians has been reported for individuals with low socio-economic status in several Ontario studies [47,48]. A recent qualitative study revealed that patients with mental health or addiction issues and service providers both expressed concerns about the impact of socio-economic status on access to primary mental health care in Canada [49]. Patients living in poverty may not have funds to cover transportation expenses when visiting a primary-care physician [49] and may have worries about housing and food availability, which impedes their ability to seek out mental health care [49]. Additionally, it is possible that these patients feel they are “undesirable” patients because of their complex care needs, unstable housing situations, physical disabilities and/or criminal records. These patients might fall through the cracks of mental health system and encounter similar barriers as those with mental disorders and/or substance abuse issues. It is also possible that these vulnerable individuals may have trouble navigating through mental health care, even when healthcare is funded and available [47,48]. However, we did find that low-income patients were more likely to use antidepressants, possibly because the ODSP covers antidepressant use for conditions unrelated to depression. Nonetheless, our results emphasize the need for the delivery of integrated care, particularly for HIV-positive patients with multiple physical and mental morbidities and for those struggling with low incomes. Not only could an integrated system provide one stop care and support for these patients, it could assist health care providers by allowing them to gain needed team support when dealing with complex issues.

Third, unlike the U.S. studies [5,6], ethnic minorities were equally likely to use mental health services in our study. However, in our study, non-native English speakers and immigrants were less likely to use mental health services. In high-income countries, immigrants are well documented to use fewer mental health services, likely due to language barriers and cultural differences [50,51]. Mental health care relies heavily on direct verbal communication between health care providers and patients. In addition, cultural barriers may cause patients and providers to view mental illness and treatment differently [52,53]. In Canada, a recent scoping review identified several major language-related barriers among Canadian immigrants preventing them from accessing mental health services [54]. The researcher showed that inadequate language skills in English or French reduced the likelihood of accessing mental health services among several ethnic groups [54]. The researcher also found that language barriers between health care providers and patients posed challenges for access to mental health services among Canadian immigrants or ethnic minorities [54].

Our study has several strengths. Our study is the first to link data between the OCS and provincial administrative databases and describe mental health service use among HIV-positive patients in Ontario. Linked data allows us to overcome the significant limitations associated with using single datasets. Our study also used utilization data and well-validated algorithms to identify patterns associated with mental health service use [35]. In addition, we performed extensive model diagnostics to ensure our final models were robust. Additionally, the diverse data provided the unique opportunity to examine various determinants for mental health services utilization. The OCS is the largest HIV medical cohort in Ontario, and participants are representative of HIV-positive patients receiving care [29]. The study is also the first to assess depression treatment adequacy among HIV-positive patients in Canada. While this variable is not a measure of care quality, results regarding treatment adequacy allow us to estimate how many patients are receiving care that meets existing guidelines.

There are also some limitations to our study. First, the screening instruments used to identify depression could lead to false positives. However, our measures have excellent agreement with DSM-IV criteria and good inter-rater agreement [32]. Second, our dataset cannot address mental health service use provided by private (non-OHIP funded) mental health professionals. However, in Canada, most patients with mental health or addiction issues seek help from family physicians (77%) or psychiatrists (55%) [33]. Many of these patients do not have sufficient income to use non-OHIP mental health services [33]. As noted above, over 70% of the study participants are unemployed or living with a disability. Thus, the participants who can access non-OHIP–funded mental health services might represent a small fraction in our study participants. Future research should investigate the use of private services. Third, primary mental health service use can be misclassified. There are potential financial incentives for HIV primary-care physicians to submit bills using HIV-related diagnostic codes rather than psychiatric ones. Fourth, while our data sources are comprehensive, we could not address some important issues, such as attitudes toward mental health care and various other reasons for not seeking mental health care.

## Conclusions

Our results show that 50% of depressed HIV-positive patients in Ontario do not receive care from primary-care physicians or psychiatrists. Approximately 51% of patients who sought help did not receive mental health care for depression in accordance with existing Canadian guidelines. Our results showed markedly unequal access to publicly funded mental health services for patients who self-identified as gay, lesbian, or bisexual, had low income or basic education only, or were immigrants or non-native English speakers.

Effective mental health policies, services, and programs are needed to address these disparities, particularly because HIV-positive patients have a longer life expectancy and could experience the negative effects of untreated depression. The HIV Strategy to 2025 proposed by the Ontario government highlights the importance of comprehensive care for HIV-positive patients, including mental health care and comorbid condition treatment. It is clear that we need effective policies to close the gaps between mental health needs and public service use. Effective policies could include developing cultural components to better treat immigrants, creating accessible services that meet the mental health needs of transgender and transsexual individuals, and exploring strategies to overcome barriers to service access, such as interpreter services for non-native English speakers, increased information/education about service availability, and system navigation services for lower income and education patients. Future research should use DSM-IV criteria for identifying major depression to verify our findings and to identify effective interventions. Additionally, there is a lack of qualitative evidence documenting the challenges facing people living with HIV and health care providers in receiving and delivering mental health care in Canada. Future research should consider conducting qualitative studies, particularly for vulnerable populations (such as LGBT individuals, patients with complex are needs, and ethnic minorities), to understand their challenges and struggles in accessing to formal and informal mental health services. Furthermore, effectiveness and cost-effectiveness are important decision-making components for the delivery of mental health services for people living with HIV in a resource-constrained health care system. Future research should consider conducting randomized controlled trials to examine the cost and effect of innovative interventions (e.g. self-management using technology) for delivering mental health services for these patients and subgroups of vulnerable populations. Moreover, although the linked data sources used in the current project are comprehensive, there are a number of limitations to the current dataset. It is limited to publicly-funded formal mental health services outcomes. In addition, although the OCS is one of the largest HIV medical cohorts in Ontario, participants mainly come from large HIV clinics in major cities and some vulnerable populations are less likely to be recruited (e.g. injection drug users, homeless patients, Indigenous people and patients from remote areas). To address these knowledge gaps, future research should consider conducting population-based studies that links multiple data sources from provincial residential mental health programs, provincial community-based mental health programs, and other support programs from AIDS services organizations. Future research should also consider collecting data for formal services use from other mental health professionals, (e.g. psychologists, social workers, nurses, and psychotherapists) and for informal services use. With these comprehensive data sources, we can then better understand the supply and demand of different types of mental health services for people living with HIV and depression.
